# Comparative Effect of Bergamot Polyphenolic Fraction and Red Yeast Rice Extract in Rats Fed a Hyperlipidemic Diet: Role of Antioxidant Properties and PCSK9 Expression

**DOI:** 10.3390/nu14030477

**Published:** 2022-01-22

**Authors:** Rocco Mollace, Roberta Macrì, Annamaria Tavernese, Micaela Gliozzi, Vincenzo Musolino, Cristina Carresi, Jessica Maiuolo, Massimo Fini, Maurizio Volterrani, Vincenzo Mollace

**Affiliations:** 1 Institute of Research for Food Safety & Health (IRC-FSH), Department of Health Science, University Magna Graecia, 88100 Catanzaro, Italy; rocco.mollace@gmail.com (R.M.); robertamacri85@gmail.com (R.M.); an.tavernese@gmail.com (A.T.); micaela.gliozzi@gmail.com (M.G.); xabaras3@hotmail.com (V.M.); carresi@unicz.it (C.C.); jessicamaiuolo@virgilio.it (J.M.); massimo.fini@sanraffaele.it (M.F.); maurizio.volterrani@sanraffaele.it (M.V.); 2 IRCCS San Raffaele Pisana, Via di Valcannuta, 88163 Rome, Italy

**Keywords:** hypercholesterolemia, red yeast rice (RYR), bergamot polyphenolic fraction (BPF), malondialdehyde (MDA), oxidative stress, proprotein convertase subtilisin/kexin type 9 (PCSK9)

## Abstract

Elevated serum cholesterol levels, either associated or not with increased triglycerides, represent a risk of developing vascular injury, mostly leading to atherothrombosis-related diseases including myocardial infarction and stroke. Natural products have been investigated in the last few decades as they are seen to offer an alternative solution to counteract cardiometabolic risk, due to the occurrence of side effects with the use of statins, the leading drugs for treating hyperlipidemias. Red yeast rice (RYR), a monacolin K-rich natural extract, has been found to be effective in counteracting high cholesterol, being its use accompanied by consistent warnings by regulatory authorities based on the potential detrimental responses accompanying its statin-like chemical charcateristics. Here we compared the effects of RYR with those produced by bergamot polyphenolic fraction (BPF), a well-known natural extract proven to be effective in lowering both serum cholesterol and triglycerides in animals fed a hyperlipidemic diet. In particular, BPF at doses of 10 mg/Kg given orally for 30 consecutive days, counteracted the elevation of both serum LDL cholesterol (LDL-C) and triglycerides induced by the hyperlipidemic diet, an effect which was accompanied by significant reductions of malondialdehyde (MDA) and glutathione peroxidase serum levels, two biomarkers of oxidative stress. Furthermore, the activity of BPF was associated to increased HDL cholesterol (HDL-C) levels and to strong reduction of Proprotein convertase subtilisin/kexin type 9 (PCSK9) levels which were found increased in hyperlipidemic rats. In contrast, RYR at doses of 1 and 3 mg/Kg, produced only significant reduction of LDL-C with very poor effects on triglycerides, HDL-C, glutathione peroxidase, MDA and PCSK9 expression. This indicates that while BPF and RYR both produce serum cholesterol-lowering benefits, BPF produces additional effects on triglycerides and HDL cholesterol compared to RYR at the doses used throughout the study. These additional effects of BPF appear to be related to the reduction of PCSK9 expression and to the antioxidant properties of this extract compared to RYR, thereby suggesting a more complete protection from cardiometabolic risk.

## 1. Introduction

Hypercholesterolemia, either associated or not to increased triglyceride serum levels, has been clearly shown to represent one of the key players in the development of atherosclerosis-associated vascular disorders including coronary artery disease and stroke [1,2]. On the other hand, a clear correlation exists suggesting that a significant reduction of low-density lipoprotein cholesterol (LDL-C) is required for both primary prevention of cardiovascular disorders and reduction of cardiovascular risk in patients with previous cardiovascular events, including myocardial infarction [3,4,5].

In the last decades, this was achieved by means of extensive use of very effective drugs such as the statins, which reduce endogenous biosynthesis of cholesterol, via inhibition of hydroxy-3-methylglutaryl-CoA reductase (HMGCoA reductase), the key enzyme of the pathway generating cholesterol from acetyl-CoA [6,7,8].

This class of drugs, due to their widespread activities in protecting vascular tissues, (anti-proliferative effects, atherosclerotic plaque stabilizing properties, reduction of vascular inflammatory biomarkers, etc.), has also been found able to reduce hospitalization and mortality in high-risk atherosclerotic patients [6,7].

Alongside their beneficial effect in the prevention and treatment of cardiovascular disorders, the use of statins is however associated to several side effects that include muscular pain, rhabdomyolysis and subsequent elevation of serum creatine phosphokinase (CPK) [9,10,11]. This seems to occur in nearly 5% of patients taking statins [12,13]. However, recent epidemiological studies revealed that nearly 30% of people stop statin treatment because of muscle aches [13,14]. Moreover, prolonged treatment with statins is also associated to increased risk of developing type 2 diabetes and neurological disorders, mostly characterized by memory loss [14]. Thus, based on the occurrence of these and other side effects, the use of statins in low-risk subjects is still controversial and alternative and more safe treatments for lowering serum cholesterol have been suggested in the last few decades, including nutraceutical supplementation by means of products able to inhibit HMGCoA reductase [15,16].

Red yeast rice (RYR) is a natural extract obtained via fermentation of white rice with the yeast *Monascus purpureus* mold that has widely been used to reduce serum cholesterol in patients [17,18]. The properties of RYR for lowering serum cholesterol remain to be clarified. However, evidence exists that RYR extract contains significant amounts of monacolin K, which is structurally identical to lovastatin, thereby suggesting a statin-like response in patients undergoing treatment with RYR [19]. To date, clinical data suggest an efficacy of RYR in lowering serum cholesterol which has been determined in a range from 14% to 24% with a satisfactory safety profile, though causality between therapy and side effects described in several studies remains to be confirmed [18,19,20]. However, a European Food Safety Authority (EFSA) Scientific Panel, in 2018, highlighted several warnings in vulnerable populations (e.g., pregnant women) and concluded “*to be unable to identify a dietary intake of monacolins from RYR, not giving rise to concerns about potentially harmful effects to health for the general population, and as appropriate, for vulnerable subgroups of the population*” [21].

Thus, based on this conclusion, it is necessary to identify better nutraceutical solutions to attenuate the potential risk of using RYR as a first line nutraceutical approach and to counteract potential side effects and uncertainties deriving from elevated amounts of monacolin K in final preparations.

Based on this preliminary evidence, the present study was addressed to evaluate the potential synergistic response in a formulation containing lower concentration of RYR either associated or not to bergamot polyphenolic fraction (BPF) a well characterized natural extract deriving from bergamot juice which has been found to produce consistent hypolipidemic response both in animal settings and in patients [15,16,22,23,24,25,26]. This was achieved in rats fed a hyperlipidemic diet.

## 2. Materials and Methods

### 2.1. Plant Material

#### 2.1.1. Red Yeast Rice (RYR)

RyR extract (NLT 3% Monacolin K) was purchased from Giellepi S.p.A (Milano, Italy).

#### 2.1.2. Preparation of BPF

*C. bergamia* Risso & Poiteau fruits were collected in the Calabrian region, from plantations that cover 90 km long costal area located between Reggio Calabria and Bianco. Bergamot juice (BJ) was obtained from peeled-off fruits by industrial pressing and squeezing. The depletion of oil fraction from juice was obtained through the stripping and the clarification by ultra-filtration; the subsequent loading on suitable polystyrene resin columns (Mitsubishi Chemical, Weekday, Japan Standard Time) that absorbed polyphenol compounds of MW between 300 and 600 Da [22]. The elution of polyphenol fractions was carried out through a mild KOH solution. Subsequently, the neutralization of phytocomplex was obtained through the filtration on cationic resin at acidic pH. Finally, it was vacuum dried and minced to the desired particle size to obtain BPF powder. The analysis of BPF powder was performed through HPLC to evaluate the flavonoid and other polyphenols content. Furthermore, in the toxicological analyses the presence of pesticides, heavy metals, phthalates and synephrine was not found (data not shown) [22]. Standard microbiological evaluation showed the absence of mycotoxins and bacteria. All procedures have been performed according to the European Community Guidelines concerning dietary supplements.

#### 2.1.3. High-Pressure Liquid Chromatography (HPLC) Analysis

High-pressure liquid chromatography (HPLC) analysis was performed on Fast 1200 HPLC system (Agilent Technologies, 5301 Stevens Creek Blvd Santa Clara, USA) equipped with DAD detector and ZORBAX Eclipse XDB-C18 column—50 mm. Two μL of sample (BPF diluted in 50% ethanol and filtered through a 0.2 μm filter) was injected eluting with a two solvent gradient of water and acetonitrile. Different gradients were used for the determination of flavonoid content or possible fumocumarin contaminants. The flow-rate was 3 mL/min and the column was maintained at 35 °C. The detector was monitored at 280 nm. Flavonoid and furocumarin pure standards were purchased from Sigma-Aldrich (Burlington, MA, USA). Brutieridin and melitidin were identified according to Di Donna [22,23]. The estimated concentration of the five main flavonoids was: neoeriocitrin (77,700 ppm), naringin (63,011 ppm), neohesperidin (72,056 ppm), melitidin (15,606 ppm) and brutieridin (33,202 ppm) [22].

### 2.2. Animal Studies

Male Wistar rats (Harlan Laboratories Ltd., Fullinsdorf, Switzerland), weighing 180–200 g, were used for the experiments. The animals were kept under stable and controlled conditions (temperature, 22 °C; humidity, 60%) with water ad libitum. Animal care was in accordance with Italian regulations on protection of animals used for experimental and other scientific purposes (D.M. 116192), as well as with the European Community guidelines [23].

The effects of BPF, or RYR on total cholesterol, LDL-C, high density lipoprotein cholesterol (HDL-C), triglycerides, malondialdehyde (expressing peroxidative damage) and paraoxonase were evaluated in Wistar rats fed a hypercholesterolemic diet composed of a standard diet (Harlan Laboratories Ltd., Fullinsdorf, Switzerland), supplemented with cholesterol 2% (pur. 95%, Sigma-Aldrich, Burlington, MA, USA), 0.2% cholic acid (min. 98%, Sigma-Aldrich, Burlington, MA, USA) and 4.8% palm oil. Moreover, the effect of these treatments on proprotein convertase subtilisin/kexin type 9 (PCSK9) levels were also studied.

The rats were divided into five groups of 10 animals each:

Group 1 (normolipidemic controls) was kept on a standard diet (Harlan) for 30 days.

Group 2 (hyperlipidemic controls) received the hypercholesterolemic diet for 30 days.

Group 3 received the hypercholesterolemic diet for 30 days; from the 1st to the 30th day, each rat was administered by gavage with BPF (10 mg/kg/rat daily, same route).

Groups 4 and 5 received the hypercholesterolemic diet for 30 days; from the 1st to the 30th day, each rat was administered by gavage with RYR extract (3 and 1 mg/kg/rat daily, respectively, same route).

During the experiment, animals were weighed weekly, and 24 h food consumption was recorded daily. On day 29, rats were individually housed in metabolic cages. At the end of the study, the animals were fasted overnight; blood samples were collected from the penile vein of the rats and serum was separated and stored at −20 °C until analyzed. The analysis of serum T-CHOL, LDL-C and HDL-C, triglycerides, MDA and paraoxonase was performed as described below.

### 2.3. Blood Measurements

At the baseline and after 4 weeks of the experimental protocol, a 12 h fasting morning blood sample was collected, processed and stored at −80 °C. All serum marker concentrations or activities were measured using classical methods and commercial assay kits, according to the manufacturers’ instructions. Assay kits for total cholesterol, LDL-C, HDL-C, triglycerides, malondialdehyde (MDA), paraoxonase and glutathione peroxidase were purchased from Novamedical S.R.L. (Reggio Calabria, Italy). All the laboratory tests were performed in a blinded manner in respect to the assigned treatment.

### 2.4. PCSK9 Measurements

For proprotein convertase subtilisin/kexin type 9 (PCSK9) assay one serum aliquot from each rat was tested by colorimetric enzyme-linked immunosorbent assay from R&D Systems (Minneapolis, MN, USA). The minimal limit of detection was 125 pg/mL, the mean intra- and inter-assay coefficient of variation was at the accepted threshold of less than 8%.

### 2.5. Statistical Analysis

In case of homogenous set of data ANOVA was performed to determine the treatment effects, and Dunnett’s test was employed as appropriate. In case of heterogeneous data, F test was carried out to determine which pairs of groups are heterogeneous. This was followed by Cochran’s or Student’s *t* tests, as appropriate. The analysis was performed by the Statistical analysis add-in component of Microsoft Excel 2007.

## 3. Results

**Figure 1:**
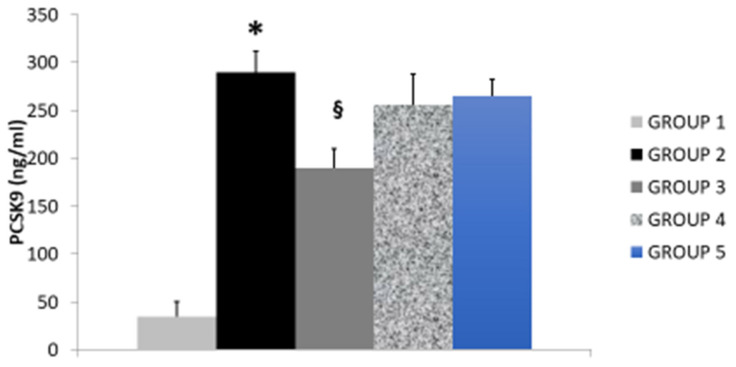
The effect of BPF (Group 3) or RYR (Groups 4 and 5) in PCSK9 serum levels in rats fed a hyperlipidemic diet (Group 2) compared to control rats (Group 1).*: *p* < 0.05 Control (Group 1) vs. Hyperlipidemic rats (Group 2); ^§^: *p* < 0.05 Hyperlipidemic (Group 2) vs. BPF or RYR (Groups 3–5). Abbreviations. BPF: Bergamot Poliphenolic Fraction; RYR: Red yeast rice; PCSK9: Proprotein convertase subtilisin/kexin type 9.

**Figure 2:**
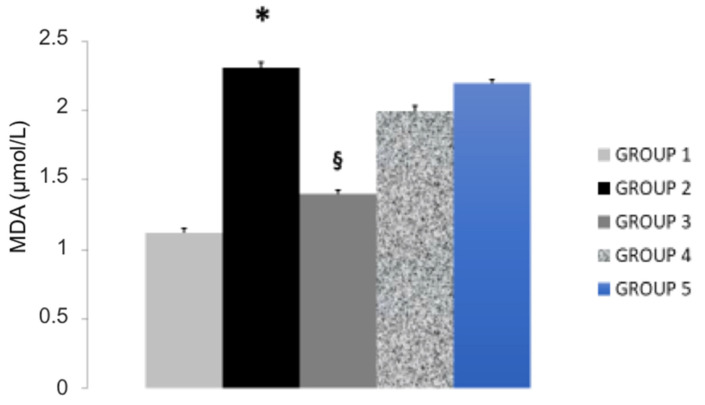
The effect of BPF (Group 3) or RYR (Groups 4 and 5) on MDA serum levels in rats fed a hyperlipidemic diet (Group 2) compared to control rats (Group 1). *: *p* < 0.05 Control (Group 1) vs. Hyperlipidemic rats (Group 2); ^§^: *p* < 0.05 Hyperlipidemic (Group 2) vs. BPF or RYR (Groups 3–5), respectively. Abbreviations. BPF: Bergamot Poliphenolic Fraction; RYR: Red yeast rice; MDA: malondialdehyde.

Administration of hyperlipidemic diet in rats (Group 2) produced, compared to control group (Group 1) an elevation of serum levels of total cholesterol, LDL-C (Table 1) and an elevation of serum paraoxonase activity, MDA and PCSK9 expression, an effect accompanied by reduction of HDL-C levels and of glutathione peroxidase, an endogenous antioxidant enzyme. These effects were found significantly attenuated by treating rats with BPF 10 mg/Kg daily for 30 consecutive days (Group 3; Table 1, Figure 1 and Figure 2). In fact BPF, according to previous evidence, significantly reduces the levels of serum total and LDL-C, an effect associated to reduced triglycerides, MDA, PCSK9 and paraoxonase activity. In addition, BPF increased HDL-C and glutathione peroxidase, as previously described.

Treatment of rats with RYR (3 and 1 mg/Kg for 30 consecutive days; Groups 4 and 5, respectively) reduced total cholesterol, LDL-C and paraoxonase (Table 1). However, when comparing the effect of RYR with BPF, we found BPF to better counteract diet-induced hyperlipidemia. Indeed, RYR produced no effect in triglycerides, HDL-C, MDA levels and glutathione peroxidase. In addition, RYR produced and very poor effects in PCSK9 when compared to BPF, thereby confirming that BPF is able to produce a better protection against diet-induced hyperlipidemia (Figure 1 and Figure 2).

## 4. Discussion

Our data show, for the first time, that BPF leads to a better hypolipidemic response when compared to RYR in rats fed a hyperlipidemic diet. In particular, our data demonstrate that BPF reduces both LDL-C and triglycerides, alongside with an elevation of HDL-C, as previously found in animal settings and patients with hyperlipidemia [15,16,24,25,26,27]. In contrast, the effect of RYR was limited to the reduction of LDL-C with no responses found in our model on triglycerides and HDL-C. On the other hand, the effect of BPF was higher to the one found in animals treated with RYR at doses used throughout the study. 

The reason for a better performance of BPF when compared to RYR still remains to be better clarified. However, antioxidant properties of BPF and its subsequent activity against overexpression of PCSK9 seem to play a role.

Accumulated evidence has shown that BPF, a polyphenolic rich extract obtained from bergamot juice, produces consistent activities in regulating serum levels of cholesterol and triglycerides acting at different levels in the lipid metabolism [15,16,24,25,26,27]. In particular, data show that BPF reduces the activity of pancreatic cholesteryl ester hydrolase, a key enzyme in the absorption of cholesterol acting at the intestinal level [24]. In addition, the lipid transfer protein system in regulated by BPF, leading to better lipid transport into the bloodstream [25]. Furthermore, it was be found that the capability of the liver to release in blood vessel non-oxydized LDL occurs in patients with hyperlipidemia and liver steatosis, thereby playing a role in the regulation of the lipoprotein traffic in hepatic tissue [27]. Finally, BPF has been found to contain several glycosylated polyphenols such as bruteridine and melitidine which have been proven to antagonize the endogenous formation of HMGCoA reductase, the key enzyme which generate endogenous cholesterol [23,24,25,26,27], thereby contributing to serum cholesterol reduction. All these activities seem to involve the formation of free radical species, as also shown by data collected in our experiments here. In fact, the effect of BPF was associated to reduction of serum levels of MDA, a well recognized biomarker of oxidative stress. This effect did not occur in animals treated with RYR.

The effect of BPF was also associated to reduced expression of PCSK9 in animals fed an hyperlipidemic diet, thus suggesting that this effect may significantly contribute in the better reduction produced by this extract when compared to RYR.

The role of PCSK9 in the regulation of cholesterol metabolism and the relative cardioprotective action has been widely studied in the last few years [28,29,30]. In particular, evidence has been collected showing that PCSK9 modulates LDL-C concentrations by binding to hepatic LDL receptors, facilitating their catabolism [28,29,30,31], thereby increasing circulating LDL-C. This is also confirmed by the evidence that newly approved PCSK9 inhibitors reduce LDL receptor degradation and lower LDL-C by >50% [32,33,34], thus offering an additional therapeutic option for patients not meeting LDL-C treatment goals with diet and maximally tolerated lipid-lowering therapy. Several recent studies also showed the key role of natural derivatives and the importance of microbiota to inhibit the PCSK9, through its transcriptional and epigenetic regulation and the subsequent up-regulation of low-density lipoprotein receptor expression, thus increasing LDL metabolism [35,36,37,38,39,40,41]. Interestingly, statin therapy itself increases serum PCSK9 levels [42], a finding that may in part explain the nonlinear relationship between statin dose and LDL-C reduction and the intra-individual LDL-C response to statin therapy [43,44]. This could explain our data with BPF and RYR in PCSK9 expression in hyperlipidemic rats. In fact, hypelipidemia is accompanied by elevation of PCSK9 [28]. This effect, in our hands, is antagonized by BPF alone but not by RYR. The reason of this discrepancy is unclear. However, being the effect of RYR mainly due to the presence of lovastatin, it is likely that an overexpression of PCSK9 may attenuate the reduction of LDL-C seen in animals treated with RYR. On the other hand, elevation of PCSK9 occurs under conditions of increased oxidative stress and inflammation [45]. This also explains the better response found with BPF compared to RYR. In fact, being BPF a powerful antioxidant action in vivo, it is likely that this extract leads to a reduced formation of free radical species in liver tissue [26], an effect accompanied by reduced expression of PCSK9. This, in turn, leads to a better efficacy of BPF compared to RYR in modulating serum cholesterol and PCSK9.

## 5. Conclusions

Our data show that BPF produces consistent benefits in reducing serum cholesterol and triglycerides compared to RYR in animals fed a hyperlipidemic diet. This better performance seems to results from a better antioxidant profile, an effect associated to reduced expression of PCSK9, and this may represent a new perspective in nutraceutical supplementation in hyperlipidemic states. Obviously, the study takes into account the potential bias and imprecision of data collected in animal models that need to be confirmed in clinical studies to be carried out in patients.

## Figures and Tables

**Table 1:** The effect of BPF (10 mg/Kg daily; *n* = 10; Group 3) or RYR (3 and 1 mg/Kg daily; *n* = 10 for each group; Groups 4 and 5, respectively) on serum T-CHOL, LDL-C and HDL-C, triglycerides, malondialdehyde (MDA) paraoxonase and glutathione peroxidase in rats fed a hyperlipidemic diet. Groups 1 and 2 (*n* = 10 for each group represent animals receiving standard or hyperlipidemic diet, respectively. Data are expressed as mean ± SD. * *p* < 0.05 Control (Group 1) vs. Hyperlipidemic rats (Group 2); ^§^ *p* < 0.05 Hyperlipidemic (Group 2) vs. BPF, RYR 3 and 1 mg (Groups 3–5). Abbreviations. BPF: Bergamot Poliphenolic Fraction; RYR: Red yeast rice; T-CHOL: Total Cholesterol; LDL-C: Low density lipoprotein cholesterol; HDL-C: High density lipoprotein-cholesterol.

Study Groups	Serum T-CHOL (mg/dL)	Serum LDL-C (mg/dL)	Serum HDL-C (mg/dL)	Serum Triglycerides (mg/dL)	Serum Paraoxonase (nmol/mL/min)	Glutathione Peroxidase (U/mL)
Group 1						
Standard diet (*n* = 10)	110 ± 12	34 ± 6	41 ± 6	145 ± 16	85 ± 6	186 ± 5
Group 2						
Hyperlipidemic diet (*n* = 10)	196 ± 14 *	117 ± 10 *	32 ± 8 *	235 ± 18 *	132 ± 8 *	175 ± 4 *
Group 3						
Hyperlipidemic diet + BPF 10 mg/Kg (*n* = 10)	154 ± 12 ^§^	73 ± 8 ^§^	46 ± 7 ^§^	175 ± 15 ^§^	102 ± 10 ^§^	214 ± 4 ^§^
Group 4						
Hyperlipidemic diet + RYR (3 mg/Kg) (*n* = 10)	164 ± 14 ^§^	83 ± 7 ^§^	38 ± 5	215 ± 19	106 ± 9 ^§^	192 ± 5
Group 5						
Hyperlipidemic diet + RYR (1 mg/Kg) (*n* = 10)	176 ± 14 ^§^	94 ± 11 ^§^	36 ± 6	222 ± 16	116 ± 5 ^§^	194 ± 6

## Data Availability

The data presented in this study are available upon request from the corresponding author.
